# Polarization-Insensitive Waveguide Schottky Photodetectors Based on Mode Hybridization Effects in Asymmetric Plasmonic Waveguides

**DOI:** 10.3390/s20236885

**Published:** 2020-12-02

**Authors:** Qian Li, Junjie Tu, Yang Tian, Yanli Zhao

**Affiliations:** Wuhan National Laboratory for Optoelectronics, Huazhong University of Science and Technology, Wuhan 430074, China; D201577615@hust.edu.cn (Q.L.); D201277599@hust.edu.cn (J.T.); D201880743@hust.edu.cn (Y.T.)

**Keywords:** Schottky photodetector, mode hybridization

## Abstract

Two types of configurations are theoretically proposed to achieve high responsivity polarization-insensitive waveguide Schottky photodetectors, i.e., a dual-layer structure for 1.55 µm and a single-layer structure for 2 µm wavelength band. Mode hybridization effects between quasi-TM modes and *sa_b_^1^* modes in plasmonic waveguides are first presented and further investigated under diverse metal types with different thicknesses in this work. By utilizing the mode hybridization effects between quasi-TE mode and *aa_b_^0^* mode, and also quasi-TM and *sa_b_^1^* mode in our proposed hybrid plasmonic waveguide, light absorption enhancement can be achieved under both TE and TM incidence within ultrathin and short metal stripes, thus resulting in a considerable responsivity for Si-based sub-bandgap photodetection. For 1.55 µm wavelength, the Au-6 nm-thick device can achieve absorptance of 99.6%/87.6% and responsivity of 138 mA·W^−1^/121.2 mA·W^−1^ under TE/TM incidence. Meanwhile, the Au-5 nm-thick device can achieve absorptance of 98.4%/90.2% and responsivity of 89 mA·W^−1^/81.7 mA·W^−1^ under TE/TM incidence in 2 µm wavelength band. The ultra-compact polarization-insensitive waveguide Schottky photodetectors may have promising applications in large scale all-Si photonic integrated circuits for high-speed optical communication.

## 1. Introduction

To meet the rising demands in optical communication, light detection and ranging (LiDAR), and nonlinear photonics, photodetectors (PDs) operating on 1.55 µm and beyond are highly desired. With the development of all-silicon integration, Si Schottky PDs based on internal photoemission (IPE) effect [1,2,3,4,5,6,7] have drawn much interest and been demonstrated in several platforms in recent decades. In such IPE-based devices, incident photons are usually absorbed by metals or silicides, and then hot carriers are produced by Landau damping [8]. The excited hot carriers are emitted through the Schottky barrier (Φ*_B_*) of the metal (or silicide)/Si interface, finally collected as photocurrent, thus achieving below-bandgap photodetection.

For IPE-based Schottky PDs, there exist three main crucial challenges, including low internal quantum efficiency (IQE), low absorption rate and weak polarization independence. Focusing on these bottlenecks, extensive studies have been carried out.

Low IQE is the main obstacle of all detectors based on the IPE effect [9]. Several solutions have been adopted to improve IQE. Some silicides with low work function to form low Φ*_B_* between silicide/Si interfaces, such as PtSi (with Φ*_B_* of 0.208 eV), have been widely used in imaging arrays [10]. However, because of their large areas and ultralow Φ*_B_*, silicide-based Schottky PDs usually suffer from a large dark current, hence stringent work conditions (such as cryogenic temperature) are required. In addition, mirror force effects [11], which are enabled by controlling the bias of Schottky PDs, are utilized to lower Φ*_B_*. Furthermore, surrounding or embedding metal structures [12,13] have also been implanted to make hot carriers reflect over multiple Schottky junction interfaces and emit to Si more easily. Moreover, it has been theoretically studied that IQE can be enhanced by introducing a surface roughness [14] of a few atomic layers for metals. In recent years, two-dimensional (2D) materials (graphene [15,16,17,18,19,20], MoS_2_ [21], black phosphorus [22,23]) have been involved in Si-based infrared PDs with superior performances comparable to commercial III–V compound and Ge-based devices. Up to date, ultrathin metals/silicides [10,24,25] are widely adopted to provide the excited hot electrons with more opportunities to emit when they bounce in the metal/dielectric interfaces.

As for the absorption efficiency improvement of Si-based Schottky PDs, several resonant structures, such as micro cavity [16,25,26,27,28,29,30], nano-antennas [31], and gratings [32,33], have been theoretically or experimentally demonstrated in some surface-illuminated devices. Micro-ring resonators [34,35,36] with high Q values are often reported to obtain narrowband absorptance enhancement for waveguide Schottky PDs. Except for the resonant structures, some of the bound modes propagating along metal/semiconductor interfaces [37,38,39,40], have been utilized in waveguide Schottky PDs. However, the *ss_b_^0^* mode (0 order bound mode whose E_y_ components are symmetric with y and x axes) with a low mode power attenuation (MPA) and weak optical confinement ability usually induces a long absorption length [37,38], while the ultra-small field profile of the *sa_b_^0^* mode (0 order bound mode whose E_y_ components are symmetric with y and asymmetric with x axes) leaves a challenge for efficient coupling from pure photonic mode [39]. Aiming to overcome these drawbacks of bound modes, mode hybridization effects between the quasi-TE mode and *aa_b_^0^* mode in plasmonic two-core waveguide [41] have been first presented and developed to achieve a high absorption rate of 95.6% theoretically within 5 nm-thick and 2.2 µm-long Au film under TE incidence for 1.55 µm. A linear array of gradient Au nanobricks employed on Si nanowire waveguide induces an absorptance of 95% for TE polarization at 1.55 µm wavelength within 830 nm of device length in theory [42]. More recently, a plasmonic photodetector utilizing supermode hybridization [43] has been experimentally demonstrated with a responsivity of 82 mA/W when a 10 V bias was applied. However, among all of the waveguide Schottky PDs, the majority hold strong dependence on the polarization of incident light, therefore limiting their applications in large scale silicon photonic integrated circuits (PICs).

It is obvious that some surface-illuminated devices with geometric symmetry are intrinsically polarization-independent [31], such as 2D nanoantenna and nanohole-based PDs. Additionally, a Schottky PD consisting of 1D multilayer grating [32] has been proposed in theory, achieving a polarization-insensitive optical absorptance of 98% at 1470 nm with the assistance of guide mode resonance. As for waveguide Schottky PDs, plasmonic silicon ridge waveguides, in which a metal film covering both the top and the sidewall of Si waveguide [44,45] are employed to achieve low polarization dependence deviation over the wavelength range of 1.2–1.6 µm. Nevertheless, to the best of our knowledge, research works on polarization-insensitive waveguide Schottky PDs have been seldom reported [44,45] over these years.

In this work, two types of waveguide Schottky PD structures are put forward, respectively for polarization-insensitive photodetection in the 1.55 and 2 µm wavelength range. In Section 2, the basic performance formulas of Schottky PDs are given. In Section 3, mode hybridization effects in two waveguide structures are analyzed and utilized to achieve high absorptance in ultrathin metal films within short lengths for TE and TM polarization. Except for the mode hybridization effects between quasi-TE mode and *aa_b_^0^* mode (0 order bound mode whose E_y_ components are asymmetric with y and x axes) which have been stated in [41], mode hybridization effects between the quasi-TM mode and *sa_b_^1^* (1 order bound mode whose E_y_ components are symmetric with the y axis and asymmetric with x axis) mode are first presented in this work. By utilizing the hybrid quasi-TE*-aa_b_^0^* modes and hybrid quasi-TM-*sa_b_^1^* modes with modified waveguide structures, our devices can obtain considerable absorptance for the incidence of TE and TM polarization, respectively. Thus, by combining with a higher IQE owing to the adoption of ultrathin metal films, a favorable responsivity can be achieved. In addition, we also use the eigenmode expansion (EME) method to make a quantitative description of the mode hybridization and absorption process, thus proving the reliability and self-consistency of the simulation results. The theoretical results of the absorption rate and responsivity spectra show broad operation wavelength ranges for these two non-resonant structures. In Section 4, the influence of the waveguide width offset on the absorption spectrum and feasibility are briefly discussed. In Section 5, performance comparisons are made between the reported polarization-insensitive Schottky PDs and this work. Our structures can possess competitive responsivity and dark current characteristics simultaneously. In addition, our design ideas may have potential applications in ultra-compact polarization manipulation and detection devices for Si PICs.

## 2. Performance Formulas

The responsivity of a Schottky PD can be obtained by

(1) $$\;R_{es}=A\eta_{i}\frac{q}{h\nu }$$

where *A* denotes the absorption rate, *q* is the elementary charge, *h* is the Planck constant and *ν* is the frequency of incident photons, *η_i_* is IQE.

The IQE can be calculated by

(2) $$\eta_{i}=\frac{1}{hv}\int_{\Phi_{B}}^{hv}P\left(E_{0}\right)dE_{0},$$

where *hν*, Φ*_B_*, *E*_0_, and *P(E*_0_*)* are incident photon energy, the Schottky barrier height, the initial energy of the hot carrier and the total emission probability from metal into a semiconductor when the excited hot carrier energy is in the range of Φ*_B_* to *hν.*

**Figure 1:**
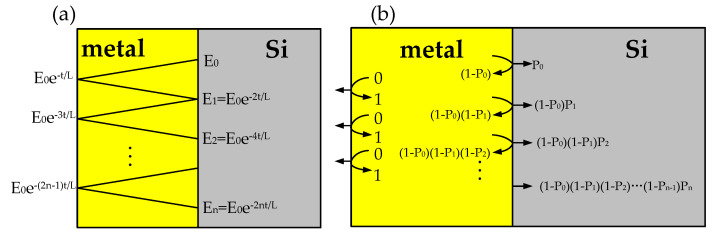
The collision model of the thin film single barrier photodetectors proposed by Scales [24]: (**a**) excess energy of a (non-emitted) hot carrier at metal/Si interface; (**b**) emission probability of a hot carrier as the number of reflections increases.

Based on the thin film single Schottky barrier model proposed by Scales [24] (which has been briefly descripted in Figure 1), *P(E*_0_*)* can be written as follows:

(3) $$P\left(E_{0}\right)=P_{0}+\left(1-P_{0}\right)P_{1}+\left(1-P_{0}\right)\left(1-P_{1}\right)P_{2}+\ldots +P_{n}\prod_{k=0}^{n-1}\left(1-P_{k}\right),$$

where $n=\frac{L}{2t}ln\frac{hv}{\Phi_{B}}$ represents the maximum reflection trips until the energy of the hot carrier is reduced to Φ*_B_*, $P_{k}=\frac{1}{2}\left(1-\sqrt{\frac{\Phi_{B}}{E_{k}}}\right)$ is the emission probability over a single Schottky barrier while the excess energy of a hot carrier is *E_k_*. After k rounds of reflection within a metal thickness of *t* and carrier attenuation length of *L*, the excess energy *E_k_* can be represented as $E_{k}=E_{0}e^{-2kt/L}$.

The dark current of Schottky PDs is closely related to the electric contact area (S) and Φ*_B_*. In Equation (4), A** is the effective Richardson constant (A** = 32 A cm^−2^K^−2^ for holes in Si), and *k_B_* is the Boltzmann constant, *T* is the absolute temperature (300 K in this work):

(4) $$I_{dark}=SA^{**}T^{2}e^{-q\Phi_{B}/k_{B}T}$$

The polarization dependent deviations (PDD) [44] can be obtained by

(5) $$\mathrm{PDD}=\frac{\left|R_{TE}-R_{TM}\right|}{\left(R_{TE}+R_{TM}\right)/2}\times 100\%,$$

where *R_TE_* and *R_TM_* denote responsivity under TE and TM incidence, respectively.

The normalized photocurrent to dark current ratio (NPDR) [46], which is a figure of merit to evaluate the detection ability, is represented as

(6) $$\mathrm{NPDR}=\frac{R_{es}}{I_{dark}}$$

## 3. Design Principle and Simulation Results

### 3.1. Dual-Layer Structure for 1.55 µm Operation

**Figure 2:**
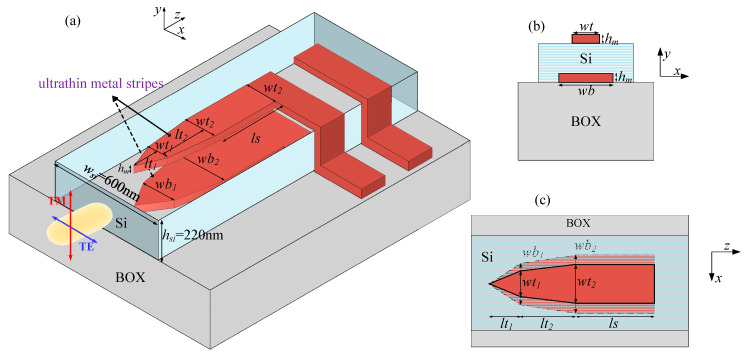
The schematic configuration of a polarization-insensitive Schottky photodetector integrated with dual-layer metal films: (**a**) the overall look; (**b**) the cross section of the hybrid plasmonic waveguide; and (**c**) the top view of the plasmonic waveguide.

In Figure 2, a hybrid photonic–plasmonic waveguide, which consists of a Si nanowire waveguide and two layers of ultrathin metal with a thickness of *h_m_* (one embedded in a Si nanowire waveguide and the other laid upwards to the Si waveguide), is schematically shown. The width and height of Si nanowire waveguide are fixed at 600 and 220 nm, marked as *w_si_* and *h_si_*, respectively. The metal stripes are both composed of two slowly varying tapers and a rectangular section.

**Figure 3:**
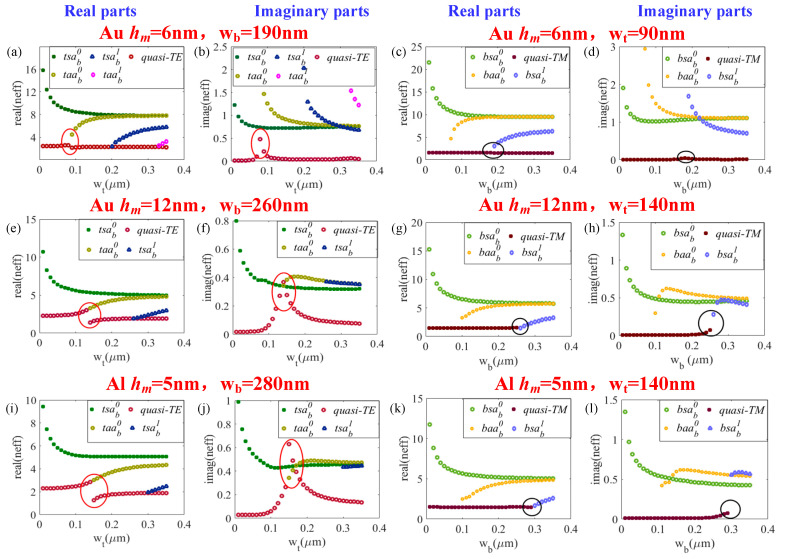
(**a**,**c**,**e**,**g**,**i**,**k**) Real parts and (**b**,**d**,**f**,**h**,**j**,**l**) imaginary parts of the mode effective indices: (**a**,**b**) versus *w_t_*, *w_b_* = 190 nm, Au, *hm* = 6 nm; (**c**,**d**) versus *w_b_*, *w_t_* = 90 nm, Au, *hm* = 6 nm; (**e**,**f**) versus *w_t_*, *w_b_* = 260 nm, Au, *hm* = 12 nm; (**g**,**h**) versus *w_b_*, *w_t_* = 140 nm, Au, *hm* = 12 nm; (**i**,**j**) versus *w_t_*, *w_b_* = 280 nm, Al, *h_m_* = 5 nm; (**k**,**l**) versus *w_b_*, *w_t_* = 140 nm, Al, *hm* = 5 nm. Other parameters: λ = 1.55 µm, *w_si_* = 600 nm, *h_si_* = 220 nm.

Owing to the complex structure of the three-core waveguide shown in Figure 2, we can make a simple classification of the modes into three categories, i.e., quasi-photonic modes with similar counterparts as photonic modes, bound modes which have a stronger electric field near the ‘top’ (‘t’) and ‘bottom’ (‘b’) metals, respectively. The following letters ‘s’ and ’a’ respectively refer to the ‘symmetry’ and ‘asymmetry’, which describe the symmetrical features of *E_y_* components with respect to *y* and *x* axes [39]. The subscripted ‘b’ represents the ‘bound’ and the superscripted number is the order of the bound mode. The mode characteristics of the plasmonic waveguide were investigated by finite difference eigenmode (FDE) solver [47] (from Mode solutions software). In the preliminary study, *w_t_* was found to have significant influence on quasi-TE modes and *t-*bound modes, while *w_b_* seems to significantly influence the effective indices of quasi-TM and *b-*bound modes. As such, we display the effective indices of quasi-TE mode and *t-*bound modes together with the varying value of *w_t_* (at a fixed *w_b_*), and quasi-TM mode together with *b-*bound modes with the varying of *w_b_* (at a fixed *w_t_*), respectively. The real and imaginary parts of mode’s effective indices as *w_t_* (*w_b_*) increases from 0.01–0.35 µm with a fixed value of *w_b_* (*w_t_*) under different metal film thicknesses are shown in Figure 3.

Mode hybridization effects [48,49,50] have been studied thoroughly among various Si waveguides with asymmetry in vertical direction over the past decade. As shown in Figure 3, there also exists a mode hybridization effect between the quasi-TE and *taa_b_^0^* mode, as circled out by the red lines, which is consistent with what has been clarified in [41]. Significantly, mode hybridization effects between quasi-TM modes and *bsa_b_^1^* modes (marked with black circles) also exist in Figure 3. In the mode hybridization regions, the imaginary parts of the effective indices for quasi-photonic modes have a significant increase, and usually the electric field of two hybrid modes are too similar to distinguish. In comparison of Figure 3b with Figure 3f, with the increase in *h_m_*, both the real parts and imaginary parts of effective indices for bound modes reduce. Moreover, the mode hybridization region “shifts” to wider metal width, and a stronger interaction occurs between the two hybrid modes, which means that the mode conversions from quasi-photonic modes to bound modes may be easier with the increase in *h_m_*. It should be noticed that a larger *h_m_* leads to a sharp deterioration of IQE [24], so a compromise consideration is required when choosing *h_m_*. Moreover, it needs to be emphasized that in plasmonic waveguide with a single-layer metal film on the top of the Si waveguide, the mode hybridization effects between quasi-TM and *sa_b_^1^* mode also exist, but the MPA of quasi-TM mode in the mode hybridization region is not ideal in our waveguide settings (*w_Si_* = 600 nm, *h_Si_* = 220 nm) to achieve high absorbance within a few micrometers. Thus, we attempted to insert another metal layer on the top of SiO_2_ to improve the MPA of the quasi-TM mode in the mode hybridization region, which have been proven helpful for the promotion of absorption under TM incidence.

In addition, a steeper rise and larger extremum of MPA for quasi-photonic mode in the mode hybridization area can be realized with a smaller *h_m_*. While, in contrast, the MPA of quasi-photonic mode outside the mode hybridization region becomes larger as *h_m_* increases (see Figure 3b,f,d,h). Particularly, quasi-TM mode is cut-off after the mode hybridization area when *h_m_* increases to some extent, as shown in Figure 3g,h,k,l.

**Figure 4:**
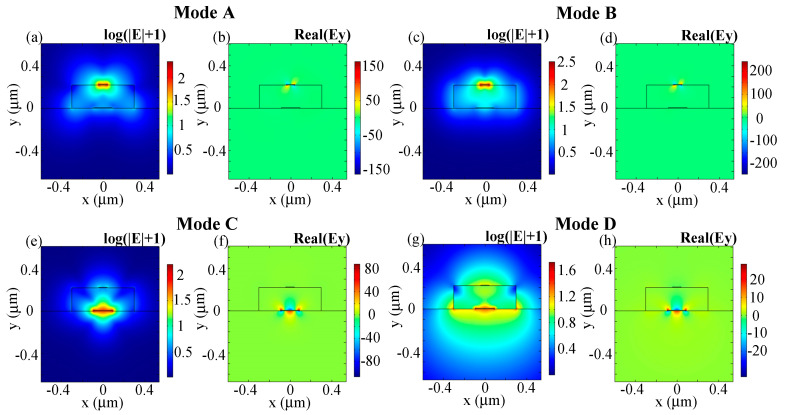
Electric field profiles of the hybrid modes: (**a**,**b**) mode A; (**c**,**d**) mode B; (**e**,**f**) mode C; and (**g**,**h**) mode D when *w_t_* = 0.08 µm, *w_b_* = 0.18 µm; (a,c,e,g) log(|E| + 1), (b,d,f,h) real(E_y_). Other parameters: Au, *h_m_* = 6 nm, λ = 1.55 µm, *h_si_* = 220 nm, *w_si_* = 600 nm.

The electric field profiles of modes A, B, and modes C, D in the dual-layer plasmonic waveguide are shown in Figure 4. Modes A and B denote the modes with a higher and lower real part of the effective index between quasi-TE and *taa_b_^0^* mode, respectively. Similarly, Mode C represents the mode which holds a higher real part of the effective index between quasi-TM and *bsa_b_^1^* mode, while mode D represents the one with the lower real part. It can be seen from Figure 4 that the electric components are comparable between modes A and B, as well as modes C and D. The E_y_ components of modes A and B are asymmetric with respect to both the *x* and *y* axes (similar to *aa_b_^0^* mode). The E_y_ components of mode C and D are asymmetric with respect to *x* axis, while symmetric with respect to *y* axis (just like *sa_b_^1^* mode).

**Figure 5:**
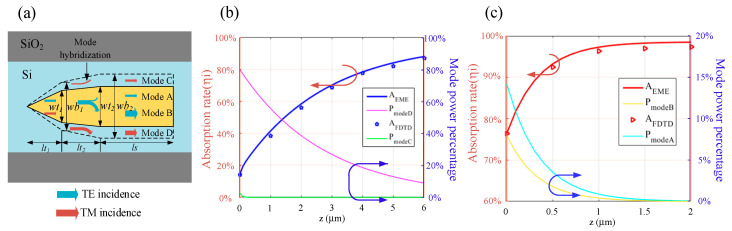
(**a**) Diagrammatic sketch of the mode propagation and conversion process under TE and TM incidence in dual-layer metal structure; (**b**,**c**) fitting results of the hybrid modes of power and absorption rate varied with the mode propagation length after the mode hybridization area, which are deduced by FDTD method and eigenmode expansion (EME) method, respectively. Structure parameters: (**b**) Au, *h_m_* = 6 nm, *wt_1_* = 0.07 µm, *wt_2_* = 0.082 µm, *wb_1_* = 0.17 µm, *wb_2_* = 0.182 µm, *lt_1_* = 0.2 µm, *lt_2_* = 0.5 µm, *ls* = 6 µm; (**c**) TM incidence. Structure parameters: Au, *h_m_* = 12 nm, *wt_1_* = 0.135 µm, *wt_2_* = 0.14 µm, *wb_1_* = 0.25 µm, *wb_2_* = 0.257 µm, *lt_1_* = 0.2 µm, *lt_2_* = 0.4 µm, *ls* = 6 µm.

Inspired by the above mode analyses, a dual-layer modified taper was proposed to achieve high efficiency polarization-insensitive absorption in ultrathin and ultra-compact metal films. The operating principle of our proposed device is schematically depicted in Figure 5a. TE mode converts to quasi-TE mode without decoupling to a pure Si waveguide, with a slowly varying taper in length of *lt_1_*. Then, the quasi-TE mode converts to quasi-TE- *taa_b_^0^* hybrid modes in a modified second taper with a specific power ratio, which is similar to [41]. The mode conversion process of TM incidence is nearly the same as that of TE incidence. As marked by red lines, the TM mode is converted to quasi-TM mode in the first taper, coupled to quasi-TM-*bsa_b_^1^* hybrid modes in the second taper, and finally absorbed efficiently in the following rectangular segment.

To make a quantitative understanding of the mode evolution process, the eigenmode expansion (EME) methods are employed to describe the power transformation between the hybrid modes we were concerned about. We also made a comparison of the finite difference time domain (FDTD) method and the EME evaluation which is deduced by Equation (7), in which *A_taper_* is the absorption rate in the first two tapers, and *A_m_* denotes the power coupled to *m*th modes at the end of the taper section (*z* = 0), *n_mi_* is the imaginary part of the *m*th mode effective refractive index, *k_0_* is the wave vector and *z* is the position in the rectangular regions along the mode propagation direction (*z* axis):

(7) $$A_{EME}=A_{taper}+\underset{m}{\sum }A_{m}\left(1-e^{-2n_{mi}k_{0}z}\right)$$

By the separated EME monitors and “power absorbed” analysis groups in the FDTD solutions software [47], the power coupled to each mode and the cumulative absorption rates at several evenly spaced *z* points along the propagation direction can be acquired easily. As plotted in Figure 5b,c, the EME evaluated results (solid lines) are nearly the same as the FDTD-derived discrete absorption rates (dotted lines), and the power percentage decrement of the hybrid modes along the *z* axis is offset by the increment of absorption rate, indicating that there are ultralow scattering loss and reflection loss, simultaneously. The results from Figure 5b,c also confirm the self-consistency and credibility of our simulations. In addition, it can be found in Figure 5c that the power in mode B and mode A are both significant after the mode conversion section in the Au-12 nm-thick case. While, the power remains mostly in mode D (quasi-TM mode in Au-6 nm-thick case) in Figure 5b, which verifies the previous statement that mode conversion between two hybrid modes is much easier when *h_m_* is larger.

In addition, dual-layer metal configurations employing various metal types and thicknesses are simulated by the 3D-FDTD method [47] (from FDTD solutions software). The corresponding optimized geometric parameters and performances are tabulated in Table 1. The theoretical responsivity of the Au-6 nm-thick configuration is ~0.12 A/W, which is an appreciable value among traditional IPE-type Schottky PDs. Owing to the high Φ*_B_* between the Al and p-Si (as high as 0.58 eV), the IQEs of Al-based Schottky PDs are relatively limited and the responsivity is much lower than that of Au/p-Si-based devices (Φ*_B_* = 0.34 eV). In contrast, also due to their high Φ*_B_*, Al-based devices have an ultralow dark current (~0.01 nA), which is three orders of magnitude lower than that of Au-based devices at least.

**Figure 6:**
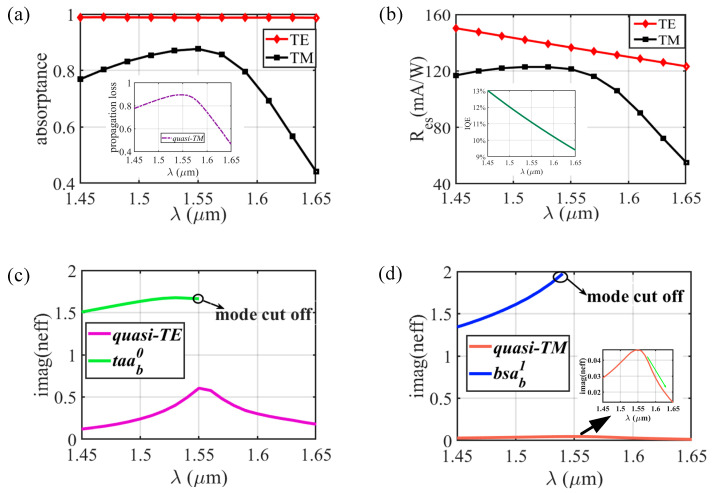
(**a**) Absorption rate and (**b**) responsivity of the dual-layer Schottky photodetector (PD) under TE and TM incidence versus wavelength. Other parameters: Au, *h_m_* = 6 nm, *wt_1_* = 0.07 µm, *wt_2_* = 0.082 µm, *wb_1_* = 0.17 µm, *wb_2_* = 0.182 µm, *lt_1_* = 0.2 µm, *lt_2_* = 0.5 µm, *ls* = 6 µm. Imaginary parts of (**c**) quasi-TE mode and *taa_b_^0^* mode, (**d**) quasi-TM mode and *bsa_b_^1^* mode in dual-layer type plasmonic waveguide versus wavelength when *w_t_* = 0.082 µm and *w_b_* = 0.182 µm.

The absorption rate and corresponding responsivity spectra of the dual-layer type Schottky PD (with an Au thickness of 6 nm) are also calculated (Figure 6a,b). Owing to the large MPA of quasi-TE mode (see in Figure 6c) and long absorption length *ls*, the A_TE_ (absorption rates under TE incidence) can be maintained at nearly 100% within a wide wavelength range. While the A_TM_ curve fits well with the power loss of pure quasi-TM mode within the propagation length of *ls* (shown in the inset of Figure 6a), proving that quasi-TM mode hardly converts to *bsa_b_^1^* mode in the whole propagation process. The MPA of quasi-TM mode reduces significantly when λ > 1.55 µm (see the inset of Figure 6d), so A_TM_ degrades as expected. Nevertheless, responsivity in the wavelength range from 1.45 to 1.65 µm is higher than 40 mA/W, which shows an improvement for most traditional Schottky PDs.

### 3.2. Single-Layer Structure for 2 µm Operation

**Figure 7:**
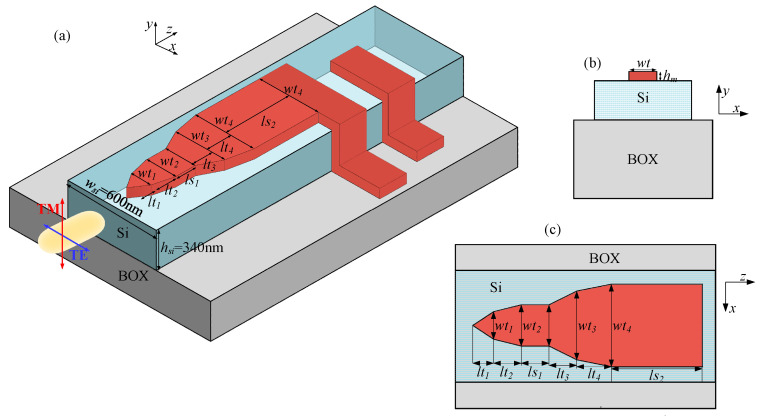
(**a**) The schematic configuration of a polarization-insensitive Schottky photodetector working in 2 µm wavelength band; (**b**) the cross section of the hybrid plasmonic waveguide; and (**c**) the top view of the absorption region.

Other than the above dual-layer structure, a modified single-layer metal stripe integrated with a Si nanowire waveguide is also developed to achieve polarization-insensitive sub-gap photodetection at a 2 µm wavelength band, as shown in Figure 7.

**Figure 8:**
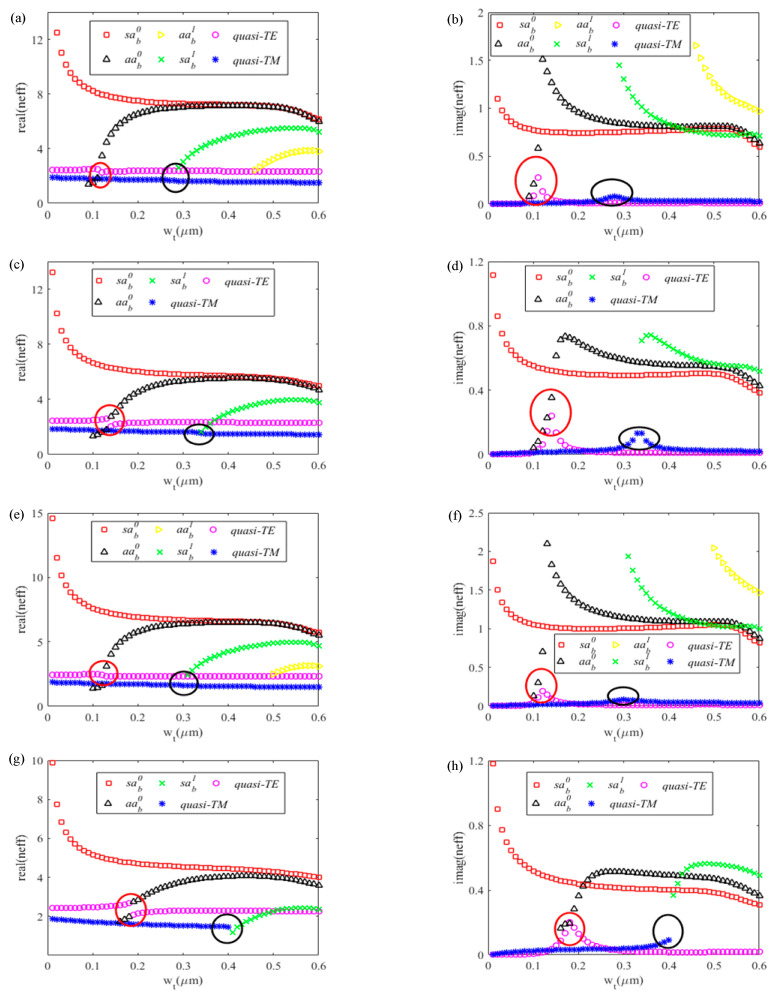
(**a**,**c**,**e**,**g**) Real parts of the effective indices versus *w_t_*; (**b**,**d**,**f**,**h**) imaginary parts of the effective indices versus *w_t_*. (**a**,**b**) Au, *h_m_* = 5 nm; (**c**,**d**) Au, *h_m_* = 7 nm; (**e**,**f**) Al, *h_m_* = 2 nm; (**g**,**h**) Al, *h_m_* = 4 nm. Other parameters: λ = 2 µm, *w_si_* = 600 nm, *h_si_ =* 340 nm.

Figure 8 shows the effective indices of the modes supported by the plasmonic waveguide as *w_t_* increases from 0 to 0.6 µm when diverse metal types and thicknesses (respectively Au-5 nm for (a) (b), Au-7 nm for (c) (d), Al-2 nm for (e) (f), and Al-4 nm for (g) (h)) are applied. In the two-core plasmonic waveguides, mode hybridization effects also exist between quasi-TM mode and *sa_b_^1^* mode, as well as quasi-TE and *aa_b_^0^* mode. The mode hybridization areas are circled out by red and black circles, respectively. In these two areas, the imaginary parts of the effective indices for quasi-photonic modes increase significantly, and then reduce. In comparison with Figure 8b,d,f,h, it can be inferred that the “interaction” between quasi-photonic modes and corresponding hybridized bound modes are stronger as *h_m_* increases, while both the real and imaginary parts of bound modes decrease. Moreover, the imaginary part curve of the quasi-photonic mode exhibits a smaller peak value in the mode hybridization region as *h_m_* increases. In the Al-4 nm case presented in Figure 8g,h, it should be noted that quasi-TM modes also turn to be “cut-off” in the posterior segment of mode hybridization regions (similar to what has been shown in Section 3.1).

**Figure 9:**
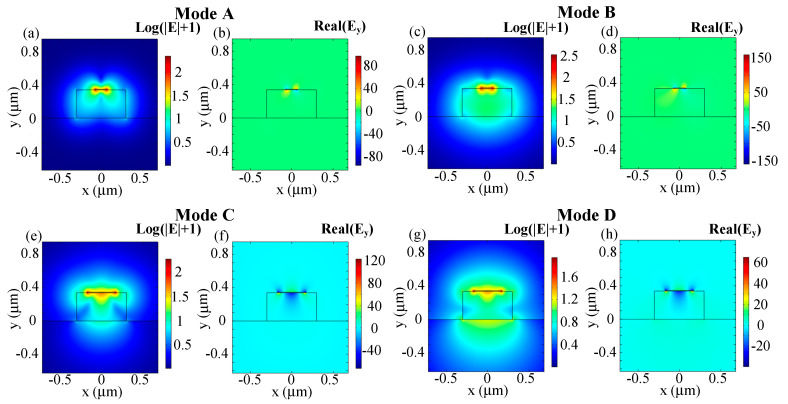
Electric field profiles of hybridized modes: (**a**,**b**) mode A and (**c**,**d**) mode B when *w_t_* = 0.14 µm; (**e**,**f**) mode C and (**g**,**h**) mode D when *w_t_* = 0.34 µm; (**a**,**c**,**e**,**g**) log(|E| + 1), (**b**,**d**,**f**,**h**) real (E_y_). Other parameters: Au, *h_m_* = 7 nm, λ = 2 µm, *h_si_* = 340 nm, *w_si_* = 600 nm.

In order to describe the mode hybridization between quasi-TE and *aa_b_^0^* modes, as well as quasi-TM and *sa_b_^1^* modes more intuitively, the normalized electric fields and vertical components of the corresponding hybridized modes (when *h_m_* of Au film is 7 nm) are plotted in Figure 9. The naming rule is identical to that employed in Section 3.1. Comparable electric field components are demonstrated between mode A and mode B when *w_t_* = 0.14 µm, and also between mode C and mode D when *w_t_* = 0.34 µm. The high resemblance of the electric field components between the hybridized modes reconfirm the existence of mode hybridization effects in the plasmonic waveguide structure. Similar to bound modes, quasi-photonic modes also hold larger electric field intensity along the metal edges and large MPA within the mode hybridization regions.

**Figure 10:**
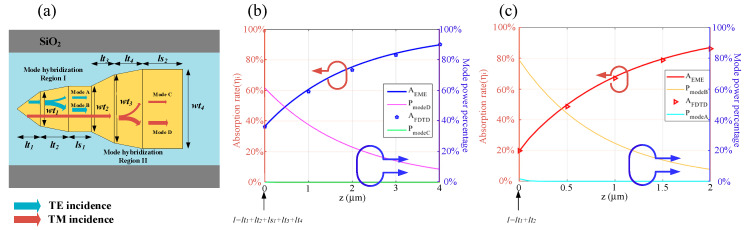
(**a**) Diagrammatic sketch of mode propagation and conversion process under TE and TM incidence in a single layer metal structure; (**b**,**c**) fitting results of the hybrid modes of power and absorption rate versus mode propagation length after the mode hybridization area. Parameters: (**b**) TM incidence, Au: *h_m_* = 5 nm, *wt_1_* = 0.09 µm, *wt_2_* = 0.11 µm, *wt_3_* = 0.25 µm, *wt_4_* = 0.28 µm; (**c**) TE incidence, Al: *h_m_* = 2 nm, *wt_1_* = 0.1 µm, *wt_2_* = 0.13 µm, *wt_3_* = 0.29 µm, *wt_4_* = 0.31 µm. Other parameters: *lt_1_* = 0.2 µm, *lt_2_* = 0.5 µm, *lt_3_* = 1 µm, *lt_4_* = 0.5 µm, *ls_1_* = 2 µm, *ls_2_* = 4 µm, λ = 2 µm.

Based on the mode analyses above, a multi-segment metal structure was put forward to achieve polarization-insensitive efficient absorption via an ultrathin metal stripe within a short device length, as shown in Figure 7. The operation principle (see also Figure 10a) is summarized as follows: for TE incidence, TE mode converts to quasi-TE mode along the first taper while wt_1_ is away from the mode hybridization area, and it may couple to hybrid modes with high MPAs in the second taper (with the length of *lt_2_*), in which mode hybridization between quasi-TE mode and *aa_b_^0^* mode exists, and finally achieve high absorptance during the propagation length of *ls_1_*. When it comes to TM incidence, quasi-TM modes are propagated and slowly weakened until the width increases to *wt_3_*, and coupled to quasi-TM-*sa_b_^1^* hybrid modes with a large MPA in the following part, eventually attenuated in the rectangular section with a length of *ls_2_*. Note that if the MPA of quasi-photonic modes under mode hybridization conditions are satisfactory enough, it is not necessary to achieve rigorous mode conversion from quasi-photonic modes to their corresponding hybridized bound modes for the purpose of absorptance promotion.

To ascertain the reliability of our simulations, the similar fitting methods which were introduced in Section 3.1 were used. The only difference is that the monitors are set in the rectangular segments with the length of *ls_1_* and *ls_2_* for TE and TM incidence, respectively. As plotted in Figure 10b,c, except for the optical power that has been absorbed, almost total residual power remains in quasi-photonic modes after the taper which functions as the mode conversion section, indicating that there is no significant mode conversion, owing to the relatively weak hybridization effects when extremely thin metal films (Au-5 nm and Al-2 nm in the above examples) are adopted.

Besides, the geometric parameters in the conditions of various metal types and thicknesses are optimized through 3D-FDTD numerical simulations, and the corresponding performances are also estimated. As summarized in Table 2, here the estimated responsivity for Au-based Schottky PDs is of 70~89 mA/W at a 2 µm wavelength band. Due to the obvious deterioration of IQE caused by the high Schottky barrier between Al and p-Si, the theoretical responsivity of Al-based devices at 2 µm is decreased by a factor of 20 compared with the result for 1.55 µm.

**Figure 11:**
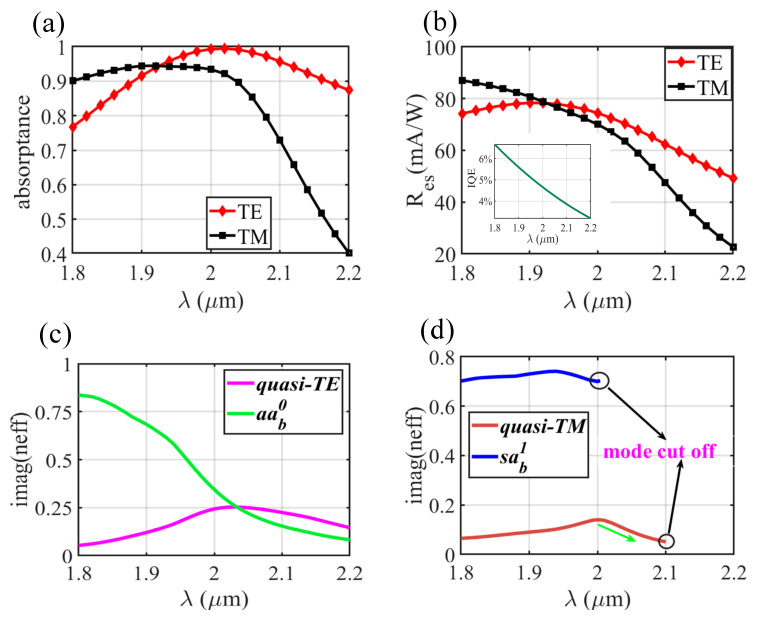
(**a**) Absorption rate and (**b**) responsivity of the single-layer Schottky PD under TE and TM incidence versus wavelength. Structure parameters: Au, *h_m_* = 7 nm, *wt_1_* = 0.1 µm, *wt_2_* = 0.14 µm, *wt_3_* = 0.31 µm, *wt_4_* = 0.338 µm, *lt_1_* = 0.2 µm, *lt_2_* = 0.5 µm, *lt_3_* = 1 µm, *lt_4_* = 0.5 µm, *ls_1_* = 2 µm, *ls_2_* = 4 µm. Imaginary parts of (**c**) the quasi-TE mode and *aa_b_^0^* mode when *w_t_* = 0.14 µm; and (**d**) the quasi-TM mode and *sa_b_^1^* mode when *w_t_* = 0.338 µm in a single-layer type plasmonic waveguide versus wavelength.

In Figure 11a,b, the absorption and responsivity spectra (Au-7 nm-thick device) in the wavelength range of 1.8–2.2 µm are plotted. The absorption rates under TE incidence (A_TE_) are almost above 80%. Due to the relatively high MPA of quasi-TE mode (see in Figure 11c) the A_TE_ is expected to be improved by increasing *ls_1_*. As shown in Figure 11d, the MPA of the quasi-TM mode starts to decrease when λ > 2 µm. Moreover, the *sa_b_^1^* mode and quasi-TM mode are respectively cut-off at 2.01 and 2.1 µm when *wt* = *wt_4_* = 0.338 µm (as 7 nm-thick Au film is adopted), so the absorption rates (in Figure 11a) for TM incidence fall drastically at a wavelength above 2 µm under our geometric settings. However, the structure exhibits a broad operation wavelength range over 300 nm (full width at half-maximum of the R_es_ spectra in Figure 11b, here the R_es_ greater than 40 mA/W are taken into account).

## 4. Discussions about Fabrication Tolerance and Feasibility

**Figure 12:**
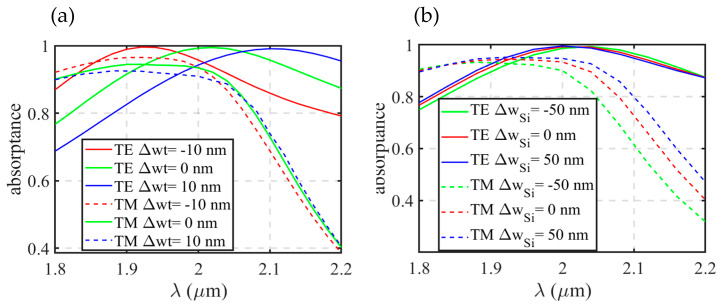
Absorption spectra of Au-7 nm-thick single layer structure with the offset of (**a**) metal width *wt*; and (**b**) Si waveguide width *w_Si_*.

In order to study the influences of fabrication error on the absorption efficiency, we performed a further simulation with the offsets of metal width (+/-10 nm) and Si waveguide width (+/-50 nm) for an Au-7 nm-thick single layer structure. As shown in Figure 12a, a +10 nm offset of metal width could result in a 100 nm red shift of the TE absorption spectrum curve. However, for TM incidence, the red shift of the absorption spectrum is less significant than that of TE incidence.

When the Si waveguide width offsets are +/-50 nm, the absorption spectra of our proposed structure are almost the same as that of original structure parameters for TE incidence (in Figure 12b). For TM incidence, the offset of the Si waveguide width would lead to a change of cutoff wavelength for the quasi-TM mode. The quasi-TM mode would be cut off at a shorter wavelength when *w_Si_* is smaller, so the absorption spectrum would drop dramatically at a shorter wavelength when a narrower Si waveguide is applied. In general, the precise control of the metal film width is very crucial, which sets a challenge for the accuracy of the pattern transfer and etch procedures.

Up to now, the ultrathin metal films (5–7 nm) with good uniformity on dielectric surfaces are extremely hard to be realized in actual fabrication. However, there have been some progresses by utilizing molecular self-assembled monolayers [51] or 2D materials [52] as adhesion layers to improve the surface wetting of the metal on the dielectric. The sputtering method [51] and chemical vapor deposition [52] are used for the growth of ultrathin metal films with good uniformity and smoothness. However, the insertion of adhesion layers would somewhat modify the Schottky interface quality. Recently, an amorphous-Si Schottky photodetector [43] has been experimentally demonstrated. The 10 nm-thick Al film is deposited by electron beam evaporator (EBE) on the surface of SiO_2_ with a surface roughness of 1.49 nm. An impressive responsivity of 80 mA/W (under 10 V bias) has been achieved in the experiment. Given the recent progresses, our proposed devices may be implanted in actual fabrication with the assistance of advanced manufacturing technology in the near future.

## 5. Summary and Future Work

In this paper, we elaborated the existing problems in the research field of Si-based Schottky PDs. By studying the mode characteristic of multi-core plasmonic waveguide, the mode hybridization effects between quasi-TM mode and *sa_b_^1^* mode are first presented, as far as we know. By utilizing the hybridized quasi-TE-*aa_b_^0^* modes, hybridized quasi-TM-*sa_b_^1^* modes through the modified tapered metal stripes simultaneously, light absorption enhancement in ultrathin metal films are achieved within short lengths (<10 µm) under both TE and TM incidence. In a dual-layer metal structure for 1.55 µm, the absorption rate of 99.6%/87.6% and responsivity of 138 mA∙W^−1^/121.2 mA∙W^−1^ are achieved for TE/TM incidence within 6 nm-thick and 6.7 µm-long Au stripes, resulting in a PDD of 12.96%. As for the single-layer metal structure for 2 µm operation, the absorption rate of 98.4%/90.2% and responsivity of 89 mA∙W^−1^/81.7 mA∙W^−1^ are achieved under TE/TM polarization incidence within 5 nm-thick and 8.2 µm-long Au stripes in theory, with a PDD of 8.5%. We also give detailed studies of the mode characteristics and obtain optimized geometric parameters based on two configurations under diverse metal types and thicknesses.

In addition, we make comparisons between some previous works on polarization-insensitive Schottky detectors and this work. Geometric parameters and device performances are listed in Table 3. Our proposal holds better responsivity owing to enhanced absorptance (~90%) resulting from mode hybridization effects and moderate IQE attributed to the adoption of ultrathin metals. Moreover, the dark currents of Au-based and Al-based devices in this work are maintained at ~100 and ~0.01 nA (under low bias), respectively. However, it should be addressed that the PDDs of our designs are not as satisfactory as expected. Actually, by reducing the A_TE_ through adjusting the metal widths artificially, an ultralow PDD also can be achieved at the expense of the R_TE_. The main contribution of this work is the observation of mode hybridization effects between quasi-photonic modes and bound modes, so only the absorption rates near the maximum under each incident polarization state are considered during the optimization processes. Meanwhile, there are relatively wide spectrum response ranges for the two types of our proposed structures, compared to some previously reported resonant-enhanced structures. We argue that the designs may have promising applications in high-density silicon PICs in the future.

Furthermore, based on the novel mode hybridization effects between quasi-photonic modes and bound modes, polarization-selective photodetection also can be flexibly realized by properly tailoring geometries, which may contribute to the detection of polarization characteristics in Si on-chip integrated circuits. In addition, the mode analyses of plasmonic waveguides may suggest the development of ultra-compact plasmonic polarization control devices, such as TE/TM-pass polarizers and polarization splitters.

Moreover, it needs to be emphasized that the theoretical responsivity in this work is inferior to the traditional photodiode based on the III–V compound or Ge/GeSn materials due to the limited IQE. To further increase the responsivity of the Schottky PDs, two-dimensional materials (such as graphene [53], MoS_2_) and multi-Schottky barrier structures are anticipated to be utilized, thus the extraction possibility of hot electrons will be boosted enormously.

## Figures and Tables

**Table 1:** Structure parameters of the dual-layer metal configuration and absorption rate at 1.55 µm simulated by FDTD under different metal types and thicknesses.

Metal	*h_m_* (nm)	*wt_1_/wt_2_* *wb_1_/wb_2_* (µm)	*lt_2_* (µm)	IQE	PS *	A_FDTD_	Res (mA/W)	PDD	I_dark_ (nA)
Au	6	0.07/0.082 0.17/0.182	0.5	11.1%	TE	99.6%	138	12.96%	98
					TM	87.6%	121.2		
	12	0.135/0.14 0.25/0.257	0.4	7.73%	TE	97.3%	93.8	13.6%	145.8
					TM	84.9%	81.8		
Al	5	0.14/0.16 0.28/0.29	0.3	1.84%	TE	98.2%	22.5	20.58%	0.015
					TM	79.7%	18.3		

* PS represents the “polarization state” of the incident light. Other parameters: *lt_1_* = 0.2 µm, *ls* = 6 µm.

**Table 2:** Structure parameters of the single layer metal configuration and performances at 2 µm.

Metal	*h_m_* (nm)	*wt_1_/wt_2_/wt_3_/wt_4_* (µm)	IQE	PS *	A_FDTD_	R_es_ (mA/W)	PDD	I_dark_ (nA)
Au	5	0.09/0.11/0.25/0.28	5.62%	TE	98.4%	89	8.5%	96.8
				TM	90.2%	81.7		
	7	0.1/0.14/0.31/0.338	4.67%	TE	99.3%	74.7	5.9%	118
				TM	93.5%	70.4		
Al	2	0.1/0.13/0.29/0.31	0.074%	TE	93.6%	1.1	1%	0.01
				TM	92.8%	1.1		
	4	0.16/0.18/0.38/0.4	0.06%	TE	97.8%	0.95	12.9%	0.013
				TM	86.2%	0.83		

* PS represents the “polarization state”. Other parameters: *lt_1_* = 0.2 µm, *lt_2_* = 0.5 µm, *lt_3_* = 1 µm, *lt_4_* = 0.5 µm, *ls_1_* = 2 µm, *ls_2_* = 4 µm.

**Table 3:** Performances comparisons with reported works on polarization-insensitive Schottky PDs.

Type	*λ* (µm)	Geometric Parameters			Performances				
		Period (µm)	*h_m_* (nm)	Area (µm^2^)	PDD	A	I_dark_ (nA)	*R_es_* (mA/W)	NPDR (mW^−1^)
Multilayer grating [32]	1.47	0.9	10	-	~0%	97%	-	1	-
Optical antenna [31]	1.43	1.3	30	-	0%	87%	-	~3	-
Plasmonic ridge WG [44]	1.2–1.6	-	10	~2.5	~5%	~90%	145	~95	655
This work	1.55	-	6	1.71	12.96%	95%	98	120	1322
	2		5	1.71	8.5%	90%	96.8	80	881
